# Rationing of Nursing Care on Example of Selected Health Care Facility

**DOI:** 10.3390/ijerph182312824

**Published:** 2021-12-05

**Authors:** Katarzyna Tomaszewska, Bożena Majchrowicz, Dorota Ratusznik

**Affiliations:** 1 Department of Nursing, Institute of Health Protection, The Bronisław Markiewicz State Higher School of Technology and Economics, 37-500 Jarosław, Poland; 2 Department of Nursing, Institute of Social and Health Sciences, East European State Higher School, 37-700 Przemyśl, Poland; bozenam4@op.pl; 3 Department of Nursing, Higher School of Health Sciences, Colegium Masoviense, 96-300 Żyrardów, Poland; dd.ratu@vp.pl

**Keywords:** rationing of nursing care, nurse, patient, BERNCA

## Abstract

Contemporary health determinants require nurses to develop new competencies and skills while performing complex tasks in all forms of health care. The problem of rationing of care is present all over the world and usually occurs when available resources are too low to provide adequate care to all patients. The most common reasons for loss of care are shortages of nurses, use of modern treatment methods, increased demand for care by a large number of patients, and greater knowledge of patients about their rights. A questionnaire survey was conducted among 295 nurses employed in hospital wards. The survey was conducted from September to December 2020 using the standardized BERNCA (The Basel Extent of Rationing of Nursing Care) questionnaire to measure the level of rationing of nursing care. The research was hampered by the sanitation regime associated with the SARS CoV-2 pandemic. Nursing care rationing is dependent on seniority and place of work. The mean total BERNCA score of the degree of rationing of nursing care was 2.58 ± 0.96 on a scale of 0 to 4 (where 0 means “no need for it” and 4 means “often”. The median score was 2.69. The higher frequency of rationing nursing care was characteristic of those working on surgical wards. The mean score obtained by them was 2.72 ± 0.86, with the median equal to 2.88. In the case of nurses employed in non-surgical wards, the scores were 2.08 ± 1.07 and 2.28, respectively. Rationing of nursing care is dependent on seniority and work location, with a higher degree of rationing of care occurring in surgical units.

## 1. Introduction

Quality of medical care is the top priority in healthcare. A large role in determining the level of quality of care is played by the actions taken by the nursing staff, and rationing is said to occur when the resources available to provide care to patients are insufficient. This is most often the case when the remaining nurses are unable to carry out the patient care plan because there are not enough nurses on duty. In addition, rationing of care is also affected by inadequate working conditions, too many patients, too little time, and excess responsibilities.

The concept of nursing rationing was first described by American nurse Beatrice J. Kalisch in 2006. The concept of loss or rationing of care occurs when required patient care is partially or completely missed, meaning that some elements of care delivery are delayed and some cannot be completed [1]. Rationing, otherwise known as rationing of care, is defined as only partially completing or not completing all of the activities that should have been done by nurses while on duty [2]. The problem of care rationing is present worldwide and usually occurs when the available resources are too low to provide all patients with proper care. The most common reasons for loss of care in addition to too few nurses are the use of modern treatments or increased demand for care by a large number of patients. Rationing of care may also be related to nurses’ attitudes, knowledge and performance of nursing activities towards some patients, resulting in unmet patients’ needs, violation of their rights and discrimination. Often, rationing of care is also related to lack of time on duty, differences in skills and resources of nursing staff [3]. The findings highlight the importance of rationing as a new systemic factor concerning patient safety and quality of care. Because at least some rationing of care seems inevitable, it is important to determine the extent of rationing to minimize negative effects on patient outcomes [4], for the reason that missed nursing care is care that is delayed, partially completed, or not completed at all [5].

Lack of care or its partial implementation is a factor that increases the risk to patients’ lives and may also lead to medical errors. Researchers’ studies show that lack of holistic care contributes to increased mortality, negative patient outcomes, increased nosocomial infections and falls, the incidence of bedsores, and decreased quality of care provided [6]. Nursing rationing refers to the abandonment or discontinuation of certain aspects of care due to limited resources, including time, staffing shortages, and misallocation of tasks. In general, it can be assumed that adverse working conditions lead to rationing of nursing interventions. In turn, limiting or omitting interventions for individual patients may affect their health [7]. Nursing rationing is also the inability of nurses to perform all patient care activities due to lack of time and resources [8].

Nursing care rationing can be assessed using the following tools: BERNCA, PIRNCA (Perceived Implicit Rationing of Nursing Care) and also the MISSCARE (The Missed Nursing Care) questionnaire proposed by Kalisch and Williams [9]. It is worth noting that one of the most important factors in rationing care is the shortage of nursing staff. According to the data maintained by the Supreme Council of Nurses and Midwives in Poland, the projected number of professionally active nurses will continue to decline, which will contribute to their shortage in the Polish market [6]. Based on continuously maintained data, the Supreme Council of Nurses and Midwives calculated the projected shortage of nurses and midwives over the next 16 years, which shows that from 2018 to 2030 there will be a total shortage of 69,886 nurses and midwives. The ratio of employed nurses per 1000 inhabitants, for the 2016 period compared to 13 countries puts Poland in last place. This indicator for Poland is 5.24, by comparison in Switzerland, it is 17.56, which places Switzerland in the first place [10].

Risks associated with working with patients during the SARS-CoV-2 pandemic, fear of infection, the unpredictability of events, feelings of helplessness, fear of performing current professional duties—these are just a few elements that nurses currently face during their work. The above elements as well as the pandemic situation may affect the level of rationing of nursing care. [11]

The aim of this study was to assess the rationing of the level of nursing care among nurses employed at a district hospital in Podkarpackie Voivodeship.

## 2. Materials and Methods

### 2.1. Research Design

In the present study, a survey was conducted among nurses employed in the wards of one district hospital in Podkarpackie Voivodeship (Poland) who were working during the survey. Its aim was to assess the level of rationing of nursing care in the studied health care unit. The cross-sectional study was conducted between September and December 2020.

### 2.2. Methods

Data was collected using the BERNCA questionnaire whose adaptation to the Polish-language version and evaluation of its reliability and accuracy in assessing the level of implicit rationing of nursing care in Poland was conducted by Uchmanowicz et al. Standard methodological requirements were followed during the translation and cultural adaptation of the English version of the questionnaire into Polish and the accuracy of the Polish version was assessed using evidence based on content and internal structure (content and construct validity). The reliability of the translated version of the questionnaire was assessed using internal consistency and inter-rater reliability. Cronbach’s alpha and inter-item correlations were used to analyze the internal consistency of the Polish BERNCA questionnaire. The mean total BERNCA score was 1.9 points (SD = 0.74) on the 0-4 scale. Cronbach’s alpha for the unidimensional scale was 0.96. The mean inter-item correlation was 0.4 (range 0.1–0.84), indicating high internal consistency. The univariate solution showed stable loadings above 0.5 for almost all items of the Polish BERNCA-R questionnaire. A study using the Polish BERNCA-R questionnaire showed that the tool is relevant and reliable for studying care rationing in groups of Polish nurses [12].

The questionnaire consists of questions about the frequency of 32 situations in which there is a need to rationalize care. The tool contains five sections that relate to patients and concern them: activities of the day, support and care, education and rehabilitation, safety monitoring, and documentation. The questionnaire contains a 5-point scale, where 0 means “no need”; 1 means “never”; 2 means “rarely”; 3 means “sometimes”; and 4 means “often”. The respondents were asked to specify how often during the last 7 days they were able to perform the activities listed in the questionnaire. The higher the score, the greater the degree of rationing of care. Additionally, the respondents completed a questionnaire containing questions about sociodemographic data: age, gender, work experience and type of ward (surgical and non-surgical), where the respondents worked.

### 2.3. Participants

The study group consisted of 295 nurses employed in the wards of a district hospital. The inclusion criteria for the study were full-time employment in hospital wards and a profession as a nurse. The exclusion criteria were lack of consent to participate in the study and employment in the hospital emergency department and operating theatre. The questionnaires were handed out to nurses in each ward and after completion were personally collected by the author of the study, who clarified any doubts on an ongoing basis.

### 2.4. Statistical Analysis

Data analysis was performed using methods of descriptive statistics and methods of statistical inference. The study group and the results obtained from the analysis of data derived from questionnaire responses were characterized using the following measures of location and dispersion: the absolute number of responses (n), structure index (%), arithmetic mean ($\bar{x}$), standard deviation (SD), median (Me), lowest value (Min) and the highest value (Max). Meanwhile, among statistical inference methods, Student’s t-test, Shapiro–Wilk test, Kruskal–Wallis ANOVA test, Mann–Whitney U test, and multiple comparisons test of mean ranks for all samples were used. Statistical hypothesis verification was performed at the significance level. Data analysis was performed using Statistica 13 statistical package (StatSoft Polska sp. z o.o., Kraków, Poland) and Excel spreadsheet from Office 365 (Microsoft Corporation, Redmond, WA, United States).

### 2.5. Ethical Procedures

The nurses’ participation in the study was voluntary and anonymous. The study was conducted in accordance with the ethical standards of the Declaration of Helsinki (64th WmA General Assembly, Fortaleza, Brazil, October 2013) and in accordance with Polish legal regulations. The application was approved by the Bioethics Committee of Colegium Masoviense of the College of Health Sciences (127/2020).

## 3. Results

The questionnaire survey was conducted among 295 randomly selected nurses employed in the wards of one district hospital in Podkarpackie voivodeship (Table 1).

Among nurses working in surgical wards, those with work experience of 21–30 years predominated (44.8%), while among nurses employed in non-surgical wards, the majority were those working in the profession for no longer than 10 years (39.7%).

### Questionnaire Results

The mean total BERNCA score of the degree of rationing of nursing care was 2.58 ± 0.96 on a scale from 0 to 4 (where 0 means “there was no need for it” and 4 means “often”. The median score was 2.69. The higher frequency of rationing nursing care was characteristic of those working on surgical wards. The mean result obtained by them was 2.72 ± 0.86, with the median equal to 2.88. In the case of nurses working on non-surgical wards, the results were 2.08 ± 1.07 and 2.28, respectively (Table 2).

A summary of the responses to each questionnaire question after recoding is provided in the next table (Table 3).

The most frequently rationed nursing activities (i.e., The most frequently rationed nursing activities (i.e., those with the highest mean scores) were: providing training and education to patients and their families (question 16), monitoring the patient’s condition as closely as needed (question 19), properly performing disinfection (question 28), developing patient care plans (question 31), taking necessary action for patients who had an unforeseen, acute, or sudden change in condition due to a long wait for a called physician (question 22), and preparing patients for upcoming tests or treatment (question 25).

The study showed that rationing of nursing care depends statistically significantly on nurses’ seniority (*p* = 0.001). In order to identify the groups of nurses between which there are the differences found above, the test of multiple comparisons of mean ranks for all samples was conducted. On its basis, we can conclude that statistically significant differences in the degree of care rationing were observed between nurses working in the profession for less than 10 years and nurses with work experience of 21–30 years (*p* = 0.002) and 31 and more years (*p* = 0.016), with nurses with longer work experience being characterized by a higher degree of care rationing. Rationing of nursing care depends statistically significantly on the nurses’ place of work (*p* < 0.001)—a significantly higher degree of rationing of care was found in surgical wards. Nurses employed in surgical wards significantly more often (*p* < 0.001) have problems with devoting enough time to support patients, educate patients and their families (*p* = 0.003), rehabilitate patients (*p* = 0.018) and monitor the patients’ conditions (Table 4).

However, there was no statistically significant difference in the frequency of problems with proper documentation of performed nursing activities between nurses working in surgical and non-surgical wards (*p* = 0.316). The age of the respondents had no effect on the level of rationing of care.

## 4. Discussion

The care provided by the nursing staff not only determines the safety of patients but also contributes to shortening the time of hospitalization and enables quicker responses to problems reported by patients. An adequate number of nurses is a prerequisite and enables them to perform nursing activities on a high level. Nowadays, however, there is a shortage of nurses who would be able to perform all assigned tasks and activities at the highest level in accordance with the current medical knowledge.

Rationing of nursing care occurs when the available number of nurses is not able to provide care and perform all assigned tasks. Nursing rationing is inextricably linked with the quality of care, which is assessed from the patient’s point of view and signifies his/her satisfaction with the care provided, professional knowledge and assistance, quick response to bothersome symptoms, information and intimacy [13].

The study showed that rationing of nursing care statistically depends on nurses’ seniority (*p* = 0.001). Respondents employed in surgical units are significantly more likely to have a problem with spending enough time educating patients and their families (*p* = 0.003). The first wave of the SARS-CoV-2 pandemic occurred during the survey implementation, which significantly affected the BERNCA questionnaire results. Respondents paid more attention to activities related to direct care and less to rehabilitation or education of patients and their families. Rationing of care concerned to a low extent the therapeutic aspects of nursing care.

In order to identify the groups of nurses between whom the above-mentioned differences occur, the test of multiple comparisons of mean ranks was performed for all samples. On its basis, we can conclude that statistically significant differences in the degree of rationing of care were observed between nurses working in the profession for less than 10 years and nurses with the length of service of 21–30 years (*p* = 0.002) and 31 and more years (*p* = 0.016), with a higher degree of rationing of care characterized by nurses with longer service. The study showed that nurses employed in the surgical wards significantly more often (*p* < 0.001) have problems with devoting enough time to support patients and do not devote enough time to educating patients and their families (*p* = 0.003).

According to our study based on the BERNCA questionnaire, a higher shortage of nurses was observed in surgical wards compared to conservative wards. As far as the patients` expectations towards nursing care are concerned, many authors noticed the discrepancies depending on the department [14]. The study performed by Jurczak et al. shows that the nursing care assessed by patients as average results from the lack of nurses on duty [15]. The results obtained are confirmed by Jankowiak, who also pointed to the insufficient number of nurses as a factor affecting the deterioration of nursing care quality [16]. The Polish Nursing Association pointed out that the most important factor determining the quality of medical services, patient and nurse safety was to ensure an adequate number of personnel [17]. Harpula et al. emphasized that the shortage of nurses is now a global problem, contributing to many consequences, including a direct threat to the patient’s life, costs associated with complications, and payment of compensation resulting from nurses’ errors [18]. Lucero also indicated an association between the lack of nurses and an increased risk of adverse events [19]. Cisek et al. in their study demonstrated that there were as many as 12–29 patients per one nurse on duty [20]. In Great Britain, the number of patients ranged from 6 to 11 [21]. Mandal emphasized that rationing of nursing care has serious consequences for the health of patients, affects job satisfaction, and increases turnover among nurses [8]. According to Schubert et al. only increasing the number of nursing staff affects the level of nursing rationing and reduces the number of tasks not performed by nurses [22].

The Gaweł’s study shows that nurses devote more time to the education of patients about the risk factors of cardiac diseases; however, education about the aim of examinations and its preparation was declared correctly by 77.8% of the nurses surveyed [23]. According to Adamska’s study, the hospitalized patients were satisfied with the nursing care and believed that the number of personnel was sufficient [24]. The study by Jurczak shows that due to the lack of an appropriate number of nurses in the surgical ward, quick responding to patients’ pain was considerably difficult [15]. In the study by Grochans, patients hospitalized in the surgical ward found that they were provided with the necessary knowledge about the way of behavior during and after surgery [25]. The lack of an adequate number of nurses contributes to imprecise and accurate procedures, which results in frequent infections [26]. The study carried out by Cisek et al. demonstrated that nurses abandoned some activities due to lack of time. The most frequent were nursing activities, administering drugs at appropriate times, performing therapeutic and therapeutic procedures and carrying out nursing activities [20]. Griffiths et al. emphasized that lack of the required number of nurses results in a lack of adequate nursing care [26]. A study conducted among nurses working in Croatia also concluded that the problem of nursing rationing affects the low quality of care offered to patients [27]. According to Zhao, the problem of care rationing can be eliminated by good cooperation in the nursing team [28]. Uchamanowicz et al. showed that there is a lack of thorough research on the reduction of care rationing in relation to the employment of support staff [12].

Our study shows that rationing of care depends significantly on nurses’ seniority and place of work. Problems with care rationing are more frequent among nurses with greater length of service and those working in surgical wards. The study conducted by Młynarska et al. shows that the level of care rationing is not affected by sociodemographic factors. However, the author demonstrated a relationship between fatigue and rationing of care. The higher the fatigue level of nurses, the higher the rationing of care [7]. The study by Cisek et al. shows that nurses, due to lack of time, were not able to devote enough time to talk to patients to calm them down [20]. Lack of care according to Recio—Saucedo significantly affects the deterioration of the quality of nursing care [29]. Tamayo et al. showed that with a better working environment there is a decrease in rationing of the total nursing care score by 0.20 units, indicating less rationing of care [30]. On the other hand, Mynaříková et al. found that healthcare administrators should make efforts to optimize nursing care and reduce gaps in care [31].

Szmidt found that nurses devoted far too little time to conversation with patients, which was associated with the instrumental performance of nursing activities [32]. In her study, Kiłoczko emphasized that the group of respondents received mostly health education services and the knowledge provided was sufficient [33]. In the study conducted by Kapala, the patients considered that the knowledge provided about the surgical procedure was very important to them [34]. Recio-Saucedo et al. emphasize that insufficient staffing results in treatment errors, patient falls, infections, or bedsores [29]. The studies carried out among nurses in England and Germany based on the BERNCA questionnaire indicated that both education and counseling were the elements of care most frequently neglected by nurses [26].

Moreover, our study shows that nurses employed in the surgical ward more often than others have problems with devoting sufficient time to monitor the patient’s condition. According to the Polish Nurses Association, an inadequate number of personnel affects patient safety, increases the number of adverse events, errors, including mortality and morbidity. Moreover, an inadequate number of nurses significantly decreases patients’ satisfaction [17]. Based on the study performed, Kózka et al. found that nurses who had fewer patients under their care rated higher the quality of their nursing activities, which included monitoring the patients’ condition every hour [35].

With the constant shortage of nurses not only in Poland but all over the world, the problem of rationing of care is and will be still present. Undoubtedly, all actions should be taken to increase the nursing staff, which will significantly influence the safety of not only patients but also the staff, reduce mortality, errors and morbidity, and most of all influence patients’ satisfaction with nursing care.

It often appears that nursing rationing is a direct response to the overwhelming demand for nursing resources in specific contexts. Therefore, discussion of resource allocation and rationing in nursing and the ethical dimensions of these issues seems timely [36]. Nursing care rationing is inevitable due to unlimited needs and limited resources. Rationing nursing care is considered an ethical issue because it requires an assessment of potential conflicts between personal and professional values [37].

### Limitations of the Study

The initial intention of the authors of this article was to survey a larger number of nurses employed in different hospitals. The epidemiological situation in our country as a result of the SARS-CoV-2 pandemic caused significant limitations in contact with a wider group of respondents, which continue to this day.

## 5. Conclusions

The study conducted among the nursing staff of a hospital in Podkarpackie voivodship (Poland) provides knowledge about the level of rationing of nursing care in surgical and non-surgical wards. The obtained results indicate that the rationing of nursing care depends on the length of service and place of work. Higher levels of care rationing are found in surgical units.

During the survey, we struggled with the first wave of the pandemic which significantly influenced the results of the BERNCA questionnaire. Respondents paid more attention to direct care activities, i.e., therapeutic, medical, or technical aspects of care, and less to rehabilitation and education of patients and their families. The primary reason for this is the isolation applied to infected individuals and the personal protective equipment used among respondents. Therefore, elements of rationing of care could not be completely avoided.

Since all current nurses employed by the hospital participated in the survey, it is possible to define which elements of the care provided included in the quality policy and nurse staffing planning should be modified or strengthened. Increasing staffing levels in accordance with current staffing standards improves the quality of care and reduces the level of rationing.

## Figures and Tables

**Table 1:** Characteristics of the study group of nurses.

Variable		Respondents *n* = 295	
		*n*	%
Sex	Man	292	98.98
	Woman	3	1.02
Age	25–35	64	21.69
	36–45	61	20.68
	46–55	129	43.73
	>55	41	13.9
Place of work (ward)	surgical	230	78.0
	non-surgical	65	22.0
Length of service	1–10	62	21.4
	11–20	60	20.7
	21–30	132	44.8
	>30	41	13.9

**Table 2:** Degree of rationing of nursing care—Overall results of the BERNCA questionnaire.

Group	*n*	%	$\bar{x}$	SD	Me	Min	Max
total	295	100.0	2.58	0.96	2.69	0	4
surgical ward	230	78.0	2.72	0.86	2.88	0	4
non-surgical ward	58	19.7	2.08	1.07	2.28	0.09	4

**Table 3:** Results of the BERNCA questionnaire survey.

Question No.	How Often in the Last 7 Days of Work...	$\bar{x}$	SD	Me	Min	Max
1	You were not able to give patients a full body bath in bed?	2.64	1.33	3	0	4
2	You were not able to perform required bedside toileting on patients?	2.58	1.27	3	0	4
3	You were not able to perform required skin care on patients?	2.53	1.24	3	0	4
4	You could not perform required oral hygiene on patients?	2.56	1.22	3	0	4
5	You could not perform the required dental hygiene tasks on patients?	2.57	1.29	3	0	4
6	You could not properly assist patients who were not eating independently with food?	2.54	1.23	3	0	4
7	You could not assist a patient with limited/impaired mobility or immobilization in moving around?	2.57	1.16	3	0	4
8	You could not reposition a patient with limited/difficult mobility or immobilization?	2.61	1.15	3	0	4
9	You could not change bedding soiled with urine, feces, or vomit for patients in a timely manner?	2.53	1.20	3	0	4
10	You could not offer emotional or psychosocial support to the patient even though in your judgment it was necessary: e.g., feelings of threat, fear, loss of independence related to the illness?	2.56	1.13	3	0	4
11	You could not have the required conversation with the patient or family?	2.60	1.15	3	0	4
12	You could not give patients enough information about upcoming tests or treatment?	2.64	1.10	3	0	4
13	You could notteach patients how to use the toilet or maintain urine, which involved the need for a diaper?	2.57	1.17	3	0	4
14	You could not teach patients how to use the toilet or maintain urine, which involved the need to insert a catheter?	2.60	1.17	3	0	4
15	You could not perform activation activities or rehabilitation interventions?	2.62	1.18	3	0	4
16	You could not provide training and/or education to patients and/or family, e.g., on insulin administration, habits, or management of specific disease symptoms (e.g., hypoglycemia or shortness of breath)?	2.73	1.13	3	0	4
17	You could not fully prepare the patient or family for the patient’s departure from the hospital?	2.59	1.29	3	0	4
18	You could not monitor the patients’ condition as closely as the doctor directed?	2.61	1.11	3	0	4
19	You could not monitor the patients’ condition as closely as you thought was required?	2.72	1.08	3	0	4
20	You were unable to observe the confused patients closely enough and it was necessary to immobilize them?	2.57	1.13	3	0	4
21	You could not observe the confused patients closely enough and it was necessary to sedate them?	2.61	1.10	3	0	4
22	You had to delay taking necessary action on patients who had an unforeseen, acute, or sudden change in condition because of a long wait for a called physician?	2.71	1.12	3	0	4
23	You were unable to administer a prescribed medication or infusion at the proper time?	2.58	1.16	3	0	4
24	You were unable to apply or change dressings for patients who required them?	2.68	1.12	3	0	4
25	You could not prepare patients for upcoming tests or treatment?	2.71	1.12	3	0	4
26	Patients who used a buzzer to call the nurse had to wait more than 5 minutes for the nurse to arrive?	2.57	1.12	3	0	4
27	You could not perform hygienic hand washing?	2.66	1.17	3	0	4
28	You could not properly perform disinfection?	2.72	1.14	3	0	4
29	You lacked time to review individual patient situations and care plans at the start of duty?	2.57	1.06	2	0	4
30	You could not properly conduct a needs assessment of newly admitted patients?	2.67	1.07	2	0	4
31	You could not develop patient care plans?	2.72	1.05	2	0	4
32	You could not properly document and evaluate the nursing activities performed?	2.63	1.10	2	0	4

**Table 4:** A multivariate comparison of statistically significant variables—job tenure and place of work of the respondents.

Variable	Group No.	Group	*n*	$\bar{x}$	SD	Me	Min	Max	*p*
Length of service	1	1–10	62	2.21	1.07	2.44	0.00	4.00	0.01
	2	11–20	60	2.52	0.84	2.66	0.00	3.68	
	3	21–30	131	2.77	0.89	2.94	0.50	4.00	
	4	>30	42	2.82	0.88	3.13	1.25	4.00	
Post-hoc ^1^	1.3; 1.4								
Ward	1	surgical	230	2.72	0.86	2.88	0.00	4.00	<0.001
	2	non-surgical	65	2.08	1.07	2.28	0.09	4.00	

^1^ Post-hoc analysis-test of multiple comparisons of mean ranks for all samples, numbers of pairs of groups that differed in a statistically significant way.
